# XX/XY System of Sex Determination in the Geophilomorph Centipede *Strigamia maritima*

**DOI:** 10.1371/journal.pone.0150292

**Published:** 2016-02-26

**Authors:** Jack E. Green, Martina Dalíková, Ken Sahara, František Marec, Michael Akam

**Affiliations:** 1 Laboratory for Development and Evolution, Department of Zoology, Downing Street, Cambridge, United Kingdom; 2 Laboratory of Molecular Cytogenetics, Institute of Entomology, Biology Centre CAS, Branišovská 31, 370 05 České Budějovice, Czech Republic; 3 Laboratory of Applied Entomology, Faculty of Agriculture, Iwate University, Morioka 020–8550, Japan; Virginia Tech, UNITED STATES

## Abstract

We show that the geophilomorph centipede *Strigamia maritima* possesses an XX/XY system of sex chromosomes, with males being the heterogametic sex. This is, to our knowledge, the first report of sex chromosomes in any geophilomorph centipede. Using the recently assembled *Strigamia* genome sequence, we identified a set of scaffolds differentially represented in male and female DNA sequence. Using quantitative real-time PCR, we confirmed that three candidate X chromosome-derived scaffolds are present at approximately twice the copy number in females as in males. Furthermore, we confirmed that six candidate Y chromosome-derived scaffolds contain male-specific sequences. Finally, using this molecular information, we designed an X chromosome-specific DNA probe and performed fluorescent in situ hybridization against mitotic and meiotic chromosome spreads to identify the *Strigamia* XY sex-chromosome pair cytologically. We found that the X and Y chromosomes are recognizably different in size during the early pachytene stage of meiosis, and exhibit incomplete and delayed pairing.

## Introduction

The centipede *Strigamia maritima* has emerged as a model system for genomic and developmental studies of myriapods [1–3]. The myriapods are an ancient lineage of arthropods, and the evolutionary outgroup to the Pancrustacea, a clade comprising insects and all crustaceans [4, 5]. They therefore occupy an important phylogenetic position to reconstruct ancestral states and to polarize the direction of evolutionary change in the Pancrustacea. One characteristic of *Strigamia* is that, in common with most other geophilomorph centipedes [6], segment number is sexually dimorphic; females of any given population will typically have a modal number of segments that is two higher than males [7]. We were interested in when this sexual dimorphism in segment number first appears during development, and this prompted us to seek ways to determine the sex of embryos.

Previous unpublished evidence suggested that the karyotype was unlikely to help. The mitotic karyotype of *Strigamia* was described by Pawel Woźnicki. He observed 2n = 16 in a male, with one relatively large pair of metacentric chromosomes, and seven pairs of smaller subtelomeric or acrocentric chromosomes. None of these chromosome pairs was obviously different in size (Pawel Woźnicki, personal communication).

However, there was one line of evidence from the *Strigamia* genome project that supported the existence of distinct sex chromosomes [1]. As part of this project, the genomic DNA of a male and two female individuals was re-sequenced and the reads mapped back onto the *Strigamia* reference genome. An analysis of the coverage of individual scaffolds in these single individuals of known sex revealed a population of scaffolds underrepresented in DNA derived from a male, but not in DNA derived from the two females [1]. This was suggestive, but not definitive, evidence for an XX/XY sex determination mechanism in *Strigamia*.

Here, we provide a detailed account of the identification and validation of the sex-linked regions in the *Strigamia* genome, and extend our previous findings by identifying and confirming the *Strigamia* sex chromosomes cytologically. The application of the sex-specific sequences reported here to provide a molecular sexing assay has already been reported [8].

## Results

### The *Strigamia maritima* karyotype is 2n = 16

**Fig 1:**
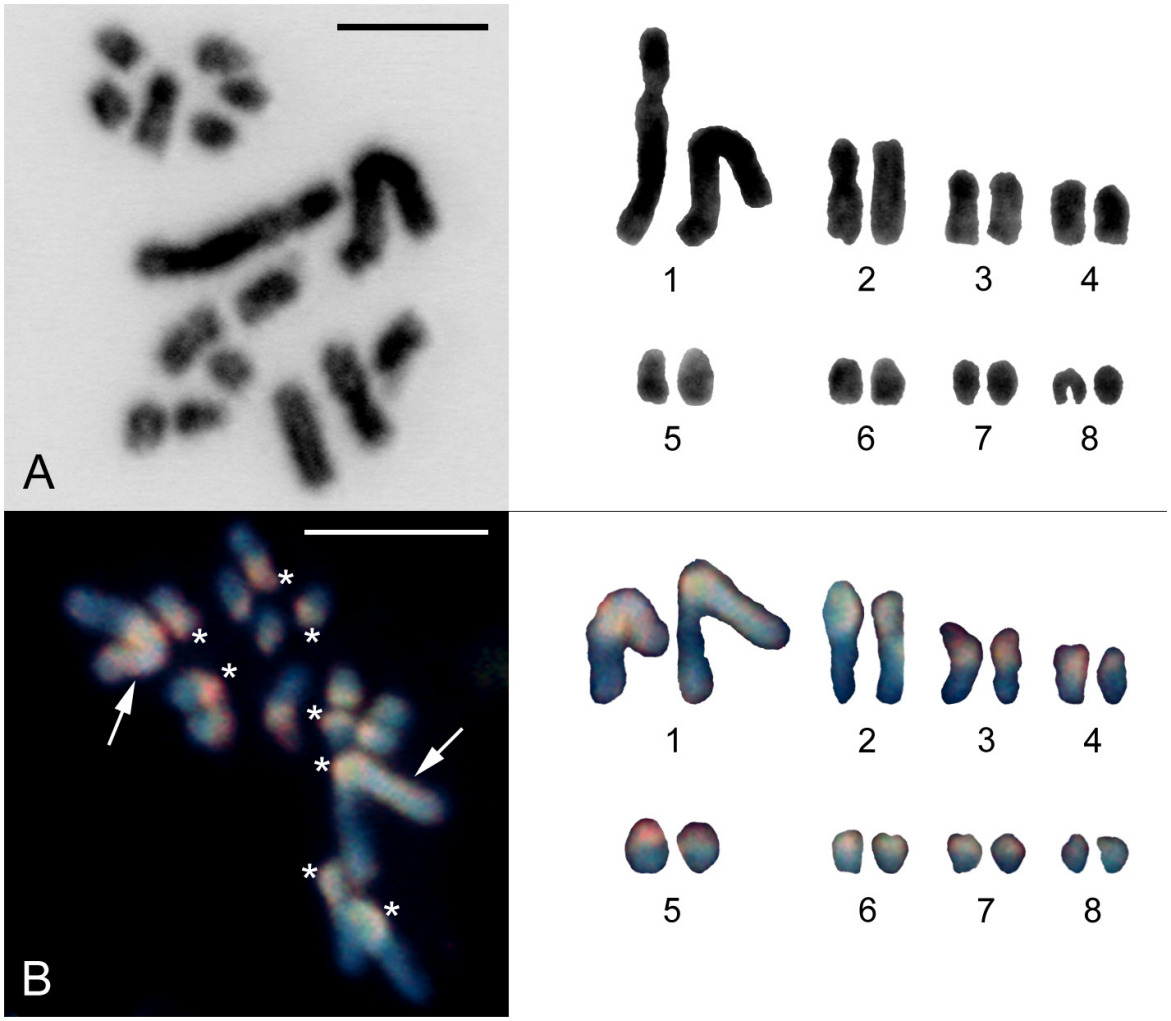
The karyotype of *Strigamia maritima*. **(A)** Inverted image of a DAPI-stained mitotic metaphase prepared from embryonic material, of which the sex was unknown (left panel). The constructed karyotype (right panel) is a representative example of karyotypes prepared from at least 10 other embryos. It shows a large pair of metacentric chromosomes and seven middle to small acrocentric pairs, gradually decreasing in size. All karyotypes give qualitatively the same result and none of them show evidence for a heteromorphic sex chromosome pair. Scale bar = 10 μm. **(B)** Comparative genomic hybridization (CGH) on a spermatogonial mitotic metaphase prepared from the testes of a sub-adult male (left panel). Chromosomes were counterstained with DAPI (blue). Female-derived genomic probe was labelled with fluorescein-12-dUTP (green) and male-derived genomic probe with Cy3-dUTP (red). Both probes highlighted one arm of the two large metacentric chromosomes (arrows) and the centromeric heterochromatin of all chromosomes (asterisks), but did not differentiate a sex chromosome pair as demonstrated in the constructed karyotype (right panel). Scale bar = 10 μm.

A previous unpublished description of the *Strigamia* karyotype had shown that 2n = 16 in a male specimen (Pawel Woźnicki, personal communication). We confirmed the *Strigamia* karyotype using mitotic spreads prepared from embryos of unknown sex (Fig 1A). All embryos examined (10/10) reproduced the same karyotype, and none of them showed evidence for a heteromorphic chromosome pair. Therefore, there was no *prima facie* evidence for a pair of heteromorphic chromosomes that might be the sex chromosomes.

We also performed comparative genomic hybridization (CGH) on spread preparations from the testes of sub-adult males. This method can differentiate X and Y, or W and Z, sex chromosomes provided that they differ sufficiently in their DNA composition [9]. However, even with CGH, we were unable to identify the sex chromosomes in mitotic spermatogonia (Fig 1B) and pachytene spermatocytes (S1 Fig).

Nevertheless, we had genomic evidence for a population of underrepresented sequences present specifically in males [1]. Therefore we decided to examine the genomic data further, and to identify a set of candidate sex chromosome-derived genomic scaffolds for validation.

## Identification of candidate sex chromosome-derived scaffolds

In an XX/XY system of sex determination, the expectation is that scaffolds located on an X chromosome will be sequenced at half the coverage in males compared with females. In contrast, unique sequences on the Y chromosome will be absent entirely from the female sequence. However, some repetitive sequences on the Y chromosome might be similar to repetitive sequences found on the X chromosome and/or autosomes, and thus be shared with females. In addition, it is likely that some fraction of Y-linked scaffolds will be misassembled, particularly if they contain repeats, and so contain a mosaic of sequences derived from X-, Y- or autosome-linked genomic regions. Thus some reads from female DNA might be assigned to scaffolds containing male-specific regions. Therefore, the prediction is that Y-linked scaffolds will not necessarily show a complete absence of mapped reads from female DNA, but that they should be sequenced at much higher coverage in males than females.

**Fig 2:**
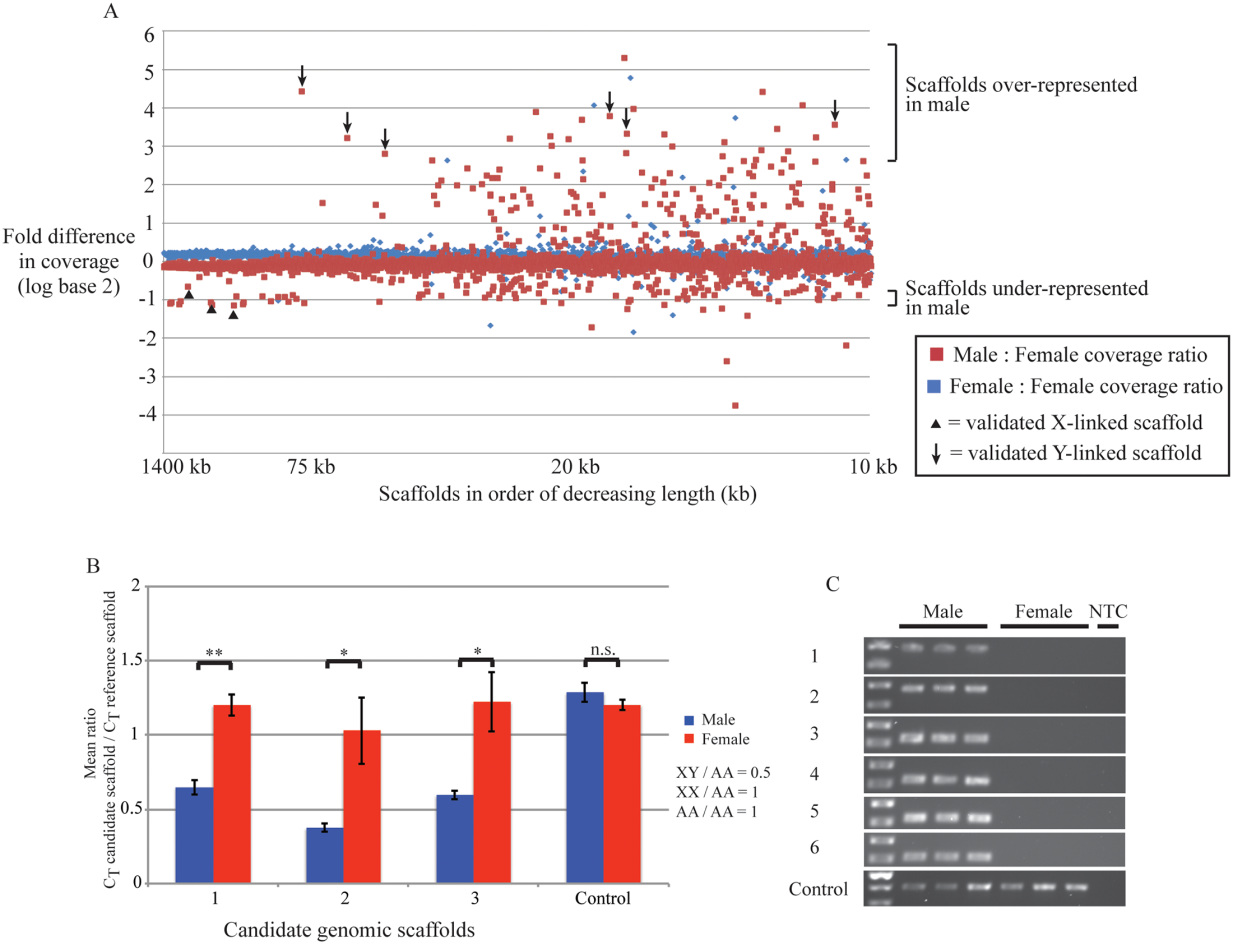
Identification and validation of X- and Y-linked genomic scaffolds. **(A)** Plot of the fold difference in coverage of each genomic scaffold between two female individuals (blue) or a male and a female individual (red), in genome re-sequencing data. Fold difference for each scaffold is shown as a logarithm to base 2. The plot reveals a number of scaffolds with different patterns of abundance in the two sexes. The regions of the plot containing scaffolds over- or underrepresented in males are indicated with brackets on the right. Only scaffolds 10 kb or more in length are shown. The scaffolds are ordered by length along the horizontal axis, with largest scaffolds on the left. Scaffolds independently validated as being located on the X or Y chromosome are indicated with arrowheads or arrows, respectively. **(B)** Quantitative PCR confirms that three tested genomic scaffolds are present at approximately twice the copy number in females as in males. The Y-axis is the mean ratio of C_T_ value of the candidate scaffold to the C_T_ value of the autosomal reference scaffold (for scaffolds 1–3: males n = 12, females n = 9; for scaffold 4: males n = 9, females n = 8). Error bars represent the standard error of the mean (SEM). Student’s t test * *P*<0.05; ** *P*<0.001; n.s. = not significant. Scaffolds 1–3 are scf7180001248200, scf7180001248049 and scf7180001247190, respectively; the control scaffold is scf7180001247533. **(C)** Identification of six male-specific genomic scaffolds. Primers designed against these 6 scaffolds only amplify a PCR product from male genomic DNA samples. In contrast, a PCR against an autosomal scaffold amplifies a product in both sexes (control). None of the primer sets amplified a product with water as the template (NTC = no template control). Scaffolds 1–6 are scf7180001247258, scf7180001245067, scf7180001243011, scf7180001247286, scf7180001247324 and scf7180001247297, respectively; the control scaffold is scf7180001247533. Original, uncropped gels are provided in S2 Fig. The raw data for this figure are provided in S2 Table.

To eliminate the noise in the coverage data produced by the large number of short scaffolds in the reference genome, we only analyzed scaffolds of at least 10 kb or longer (a set containing substantially over half the genome—the contig N50 of the assembly is 24.7 kb). For this set of scaffolds, we examined two ratios. First, the ratio of coverage of each scaffold in a male sample relative to a female sample, and second, the ratio of coverage in two female samples (Fig 2A). It is clear that the vast majority of scaffolds show no major difference in coverage between the two females, or between males and females, as expected if most of the genome is autosomal. However, it is also clear that there are at least two populations of scaffolds with distinct patterns of coverage in male and female samples (Fig 2A; regions of plot indicated with brackets).

First, there is a population of scaffolds present at about half the abundance in males compared with females (Fig 2A; lower bracket). There are 69 such scaffolds of 10 kb or more in length, with a fold difference in coverage between -0.75 and -1.25 log base 2, totalling 5000 kb of sequence (S1 Table). Note that this ratio trends towards a precise two-fold difference (log base 2 ratio of -1) as the scaffolds get longer, reflecting the more precise estimates of coverage for the long scaffolds. We took this population as a set of candidate X-linked scaffolds, and tested for quantitative differences in copy number between the sexes.

Second, there is a population of scaffolds substantially over-represented in males compared with females (Fig 2A; upper bracket). The identification of male-specific scaffolds is complicated by the issue of repetitive elements that are common to the X and Y chromosomes and autosomes. Such repetitive regions may cause reads from female DNA to be mis-assigned to scaffolds in the genome assembly containing male-specific regions. Nevertheless, the prediction is that Y-linked scaffolds should be sequenced at much higher coverage in males than females. Therefore we selected candidate male-specific scaffolds for validation using the somewhat arbitrary cutoff that they should show, in the genome re-sequencing data, a 7-fold, or higher, ratio of coverage in males over females. This cutoff identified 24 scaffolds 10 kb in length or larger, totalling approximately 512 kb of sequence (S1 Table). We took this population as a set of candidate Y-linked scaffolds, and tested whether unique sequences in these scaffolds were present only in males.

### Validation of three X-linked scaffolds

We selected for validation three of the longest scaffolds underrepresented in males relative to females (scf7180001248200, scf7180001248049 and scf7180001247190). After confirming that these scaffolds generated a PCR product in both male and female genomic DNA samples, we next tested these scaffolds for differences in copy number between the sexes using quantitative real-time PCR (qPCR). The expectation is that any sequences located on an X chromosome will be present at twice the copy number in females (XX) compared with males (XY) [10].

The qPCR confirmed the expectations from the analysis of scaffold coverage. We find clear evidence that at least these three underrepresented scaffolds are present at half the copy number in males compared with females (Fig 2B; scaffolds also indicated on Fig 2A with arrowheads). This is most likely due to the location of these scaffolds on an X chromosome in an XX/XY sex determination system. A second region on the reference *Hox* scaffold was tested as a control. This control region was located more than 240 kb away from the region against which the reference primers were designed. As expected, this control region showed no significant difference in abundance between male and female individuals (Fig 2B).

### Validation of six male-specific scaffolds

We had an initial set of 24 candidate Y-linked scaffolds to validate. To try and avoid the problem of repetitive elements, we searched for sequences in these scaffolds that were unique within the assembled genome—i.e. regions of sequence that only return hits to their constituent scaffold, and not to other scaffolds in the genome (using a sliding window of 1 kb, see Methods). Only 7 of the 24 scaffolds contained any unique regions at all. The remainder contained repetitive sequences within every 1 kb window. We designed PCR primers within the unique regions of 6 of these 7 scaffolds. For these 6 scaffolds, the primer sets amplified a PCR product only from male genomic DNA (Fig 2C; scaffolds also indicated on Fig 2A with arrows). To control for the quality of the female genomic DNA, we designed PCR primers against the scaffold containing the *Hox* cluster, which shows no significant difference in coverage between the sexes, and confirmed that these primers amplify a product in both sexes (Fig 2C). Therefore, we have found at least 6 male-specific scaffolds in the *Strigamia* genome. This is most likely due to the location of these scaffolds on a Y chromosome.

Note that even those few "unique" male-specific fragments identified in the above assay must contain short repeats derived from repetitive regions that are not present in the final genome assembly. When we tried to use these probes for in situ hybridization (see further comment below), they hybridized at many sites in the genome. However, the paired PCR priming site sequences themselves must be present only on the Y chromosome.

### Cytological identification of the *Strigamia* XY chromosome pair

Having found genomic and molecular evidence for a set of X and Y chromosome-derived scaffolds, we wanted to confirm the existence of X and Y chromosomes cytologically. To achieve this, we performed fluorescent in situ hybridization (FISH) against chromosome spreads using X- and Y-chromosome derived DNA probes. We attempted to isolate mitotic spreads from adult centipedes of known sex, but mitoses were rare in the adults available, and difficult to prepare. We therefore used embryos, which contained abundant mitoses, but were of unknown sex, and male testes, which contained meiotic chromosomes.

Attempts to use the short Y-specific sequences as probes were unsuccessful; these were too short to give any clear single copy in situ signal above a dispersed background of hybridization.

**Fig 3:**
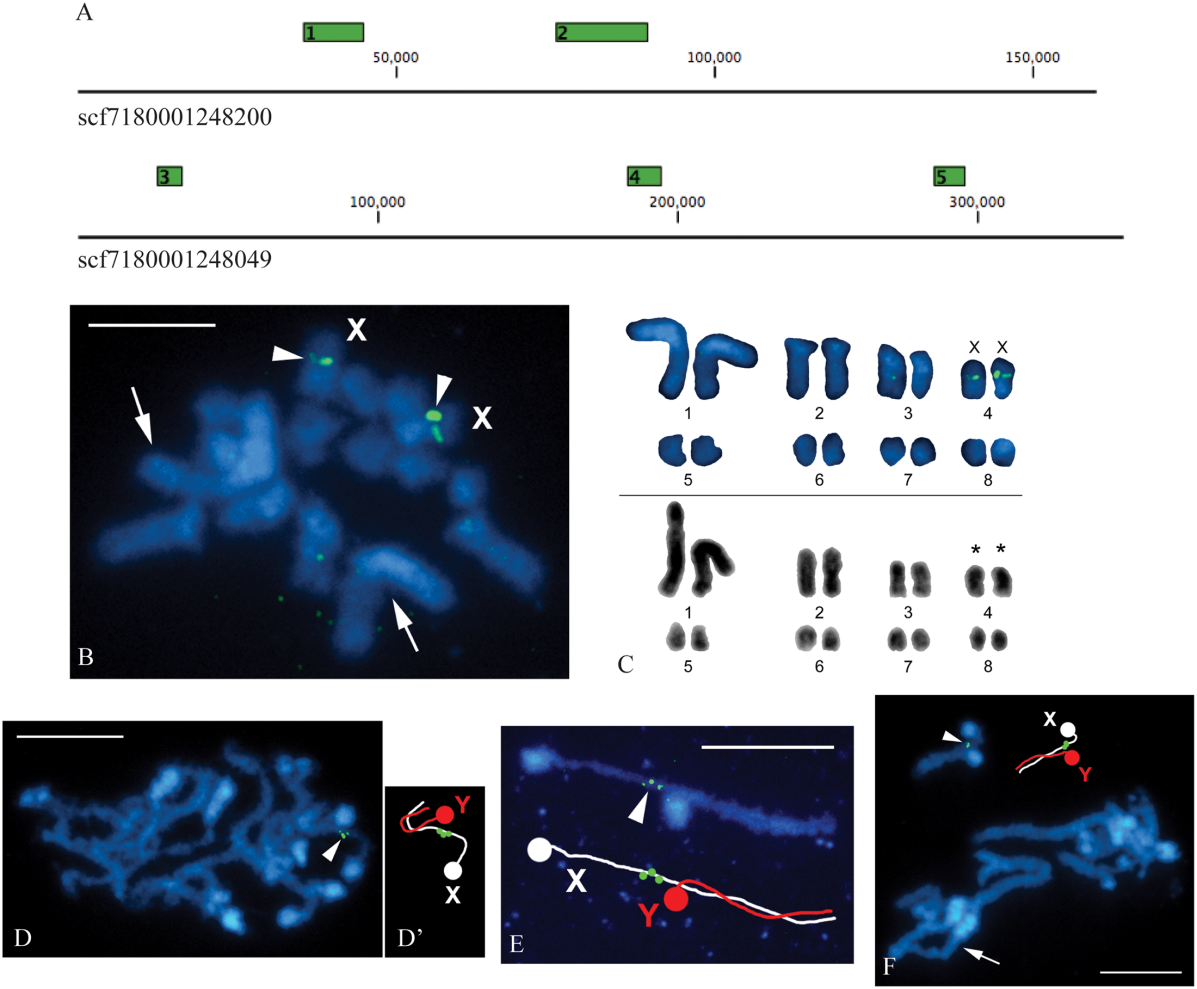
Identification of the X chromosome in the *Strigamia* karyotype by FISH with a set of X-chromosome derived DNA probes. **(A)** The relative positions and sizes of the five PCR fragments, distributed on two different X-linked scaffolds, which were used to produce the X-probes. Two of the fragments are located on scf7180001248200 and three on scf7180001248049. The genomic distance between these scaffolds is not known. The black line depicts the scaffold. The numbers above the line indicate the number of bases, starting at 1 on the left hand side. Green boxes represent the length and relative position of the PCR products, numbered arbitrarily from 1 to 5. **(B)** A mitotic metaphase chromosome spread prepared from a single embryo. Hybridization signals of the X-probes identify a middle-sized element in the *Strigamia* karyotype as the X chromosome. As there are two chromosomes with the X-probe signals, we infer that this chromosome spread is derived from a female embryo (XX). **(C)** Two *Strigamia* karyotypes constructed from the mitotic metaphases of embryonic cells. They are derived from different embryos. Upper panel: karyotype derived from the female metaphase shown in (B). Lower panel: karyotype derived from an inverted image of a DAPI-stained metaphase of unknown sex. It is the same as that shown in Fig 1A. We infer that the pair of sex chromosomes represents the 4^th^ pair of chromosomes by size (asterisks). (D, E, F) Meiotic chromosome spreads, prepared from sub-adult male testes. **(D)** Late zygotene complement showing a clump of incompletely paired bivalents. The X-probes label the longer chromosome of a partially paired bivalent, as schematically illustrated in (D’). We thus infer that this is the X chromosome, and that the other shorter chromosome, without hybridization signals, is the Y chromosome. The X and Y chromosomes are only paired at the distal part of the X chromosome, with a large proximal part unpaired. **(E)** A particularly clear and well-spread XY bivalent at a similar stage to (D). It shows hybridization signals of X-probes on the unpaired proximal part of the X chromosome, while the Y chromosome is completely paired except for the DAPI-highlighted centromere (see schematic drawing below the XY bivalent). **(F)** Pachytene complement showing 8 bivalents, each with DAPI-highlighted centromeric chromatin. X-probe hybridization signals are visible on the unpaired segment of the longer chromosome, near the centromere (see schematic drawing on the right-hand side). The X and Y chromosomes now appear almost equal in length in the bivalent. Scale bar is equal to 5 μm in (B) and 10 μm in (D, E, F). Chromosomes were counterstained with DAPI (blue). Arrowheads indicate hybridization signals of the digoxigenin-labelled X-probes (green); arrows indicate a pair of the largest chromosomes (B) or the largest bivalent (F).

We were, though, able to design a set of X-linked probes that contained minimal repeat sequence, so as to minimize hybridization to other, non-sex-linked regions of the genome. Using a sliding window of 10 kb, we searched the three validated X-linked scaffolds for unique regions. Having found long stretches of unique sequence in each X-linked scaffold, we designed multiple primer pairs in these regions in order to generate PCR fragments spanning the unique regions. In total, we successfully amplified five PCR fragments, two on scf7180001248200 and three on scf7180001248049, covering a total of 54.8 kb of non-consecutive sequence. The relative sizes and genomic positions of these fragments are shown in Fig 3A. These PCR fragments were labelled with digoxigenin and used as a cocktail of X-linked probes for FISH. We performed FISH against mitotic metaphase spreads prepared from single embryos (of unknown sex) and against meiotic spreads prepared from the testes of sub-adult males.

We identified a number of mitotic spreads with positive signals on a pair of middle-sized chromosomes (Fig 3B; arrowheads). We infer that this is a pair of X chromosomes, and thus that the spread is derived from a female embryo. We ordered the FISH-labelled chromosomes by size, and compared this with a previously constructed mitotic karyotype, derived from a different embryo (same karyotype as shown in Fig 1A). Through this comparison, we infer that the X chromosomes represent the 4^th^ pair of chromosomes by size (out of eight pairs of chromosomes).

Meiotic spreads prepared from sub-adult male testes contain late zygotene/pachytene stages, in which the chromosomes are lining up with each other to form homologous chromosome pairs, or bivalents. If *Strigamia* males possess an X and Y chromosome, the expectation is that only one chromosome of the sex chromosome bivalent, the X chromosome, should be labelled with the X-probes.

The meiotic spreads that we have examined match this expectation. We have identified a number of meiotic prophase I nuclei in which, in one of the eight bivalents, positive signals are located on only one of the two chromosomes (Fig 3D–3F). In some zygotene/early pachytene complements, in which the chromosomes are incompletely paired, a middle-sized bivalent can be seen in which the two chromosomes are of unequal length. The X-probes label an unpaired segment of the longer chromosome, the presumptive X chromosome (Fig 3D and 3E). We infer that the other shorter chromosome, without hybridization signals, is the Y chromosome. A particularly clear example is shown in Fig 3E, where the XY bivalent is well spread and separated from the other bivalents. It can be seen that X and Y chromosomes are only paired along part of the X chromosome. A large part of the X chromosome remains unpaired, while it appears that the Y chromosome is completely paired except for the terminal centromeric heterochromatin.

In presumably later pachytene complements, we find an approximate equalization of the lengths of the two sex chromosomes in the XY bivalent (Fig 3F). In support of our inference that this represents the XY bivalent, we note that similar phenomena—delayed pairing and length equalization between sex chromosomes of different sizes—is known to occur in a number of other species [11, 12].

Note that it is not surprising to find that the X and Y chromosomes are visibly different in length during the pachytene stage of meiosis, but that such size differences are not detectable in the mitotic karyotypes of these very small chromosomes (compare Fig 3B, 3C and 3D, 3E). This is because pachytene chromosomes are significantly de-condensed and can appear much longer than the highly compact mitotic chromosomes [13, 14]. This means that, within the resolution limit of conventional fluorescence microscopy, differences in size are much easier to detect at this stage.

## Discussion

Taking together the genomic, molecular and cytological evidence, we conclude that *Strigamia* has an XX/XY chromosome system of sex determination, with males being the heterogametic sex. To our knowledge, this is the first report of sex chromosomes in any geophilomorph centipede. In addition, we note that the evidence from the comparative genomic hybridization (Fig 1B and S1 Fig) and the degree of pairing observed during meiosis (Fig 3D and 3E), suggest that the *Strigamia* X and Y chromosomes are poorly differentiated, and may therefore be evolutionarily young sex chromosomes.

It is clear from the difficulty that we had in identifying unique sequences in the male-specific scaffolds that even the best assembled parts of the *Strigamia* Y chromosome comprise mostly repetitive DNA. The identification of such repeat-rich, male-specific regions in genome sequences assembled from short read data, with no or little chromosome-level information, is a general problem. Here, we used a two-step approach to overcome these difficulties. First, we identified scaffolds over-represented in independently sequenced male DNA relative to female DNA, and focussed on long scaffolds (10 kb or more in length) for which estimates of coverage are more precise. Second, within each scaffold we selected regions for PCR validation that were unique within the assembled genome. This strategy enabled us to identify six *Strigamia* scaffolds containing male-specific sequence. A conceptually similar but distinct approach was used to identify Y-linked scaffolds in *Anopheles* mosquitoes [15].

It is likely that much of the *Strigamia* Y chromosome is not represented in the assembled genome, but remains unidentified in the 42% of repetitive sequence reads that could not be assembled [1]. It is therefore difficult to make any estimate of the fraction of the genome that is contained in the Y chromosome. With regards to the X chromosome, the 5 megabases of sequence that we identify as significantly underrepresented in males as compared to females represents approximately 3% of the assembled genome length (176 Mb) and under 2% of the total genome, estimated to be 290 Mb [1]. This is an underestimate of the size of the X-specific region, as short scaffolds cannot be identified reliably as X-linked by sequence coverage.

In general, little is known about the chromosome biology of centipedes [16, 17]. From the available data, centipedes exhibit a diversity of karyotypes, with chromosome number varying from 2n = 14 to 54 across the class [16–19]. The mechanism of sex determination is determined for several species in two centipede orders, Scutigeromorpha and Scolopendromorpha. In these taxa, XX/XY male heterogamety is reported [20–22]. In all centipedes examined, no X0 species, or species with female heterogamety (WZ/ZZ), has ever been described. Multiple sex chromosome mechanisms have been described in members of the genus *Otocryptops* (Scolopendromorpha), with chains of X and Y chromosomes visible during meiosis [23–25]. However, by comparison with closely related taxa, it is clear that these variations are derived from an ancestral XY system [16, 26].

Outside of the centipedes, the mechanism of sex determination is determined for several species of millipedes and pauropods [27–30]. In all millipedes investigated to date, the male is heterogametic with the vast majority having X and Y chromosomes, but a handful of cases are reported with an X0 mechanism [27–29]. Both XY and X0 mechanisms are described in pauropods [30]. It is therefore likely, given the occurrence of X and Y sex chromosomes in at least three orders of centipedes (Scutigeromorpha, Scolopendromorpha and now Geophilomorpha) [16–19], and their occurrence in two other myriapod lineages (millipedes and pauropods), that the ancestral state for the centipedes is an XX/XY system of sex chromosomes.

## Materials and Methods

### Genomic DNA extraction

Adult centipedes were collected from the field at a stretch of shingle bank beach near Brora, northeastern Scotland (coordinates: 57°59'N, 03°55'W) as described previously [31]. Once returned to the lab, individual adults were sexed according to morphological criteria [8, 32]. The final leg-bearing segment and the genital segments were removed by dissection from female adults. This was to ensure complete removal of the spermatheca from female specimens, to avoid any contamination with male DNA. Adults were snap frozen in liquid nitrogen and stored at -80°C until processing. Genomic DNA (gDNA) was extracted from male and female specimens with the Genomic DNA Buffer Set and Genomic-tip 20/G (Qiagen) according to the manufacturer’s instructions.

### Identification of candidate sex-linked scaffolds

The *Strigamia maritima* reference genome (release Smar_1.0) is available at www.ncbi.nlm.nih.gov/assembly/322118/. The genome was assembled from a mixture of both male and female samples. The reads for the re-sequenced individuals used to identify the candidate sex chromosome-derived scaffolds are stored at the NCBI Sequence Read Archive (females A and B—SRA accessions SRX326837 and SRX326839, respectively—and male J—SRA accession SRX326841). The mapping of reads back to the *Strigamia* reference genome was completed by the *Strigamia* genome project [1]. For each re-sequenced individual, the coverage for each scaffold was calculated as the number of reads that mapped back to that scaffold in the reference genome, multiplied by the length of the sequence reads, and divided by total scaffold length. Individuals A and B had sequence reads of 95 bp, and individual J of 125 bp. Finally, for each individual, scaffold coverage was normalized to the mean coverage across all scaffolds. The ratios of scaffold coverage in different individuals were calculated, and the plots generated, in Microsoft Excel. The raw data is provided in S2 Table.

### Quantitative real-time PCR

Relative quantification of genomic scaffolds between the sexes was performed on a 480 LightCycler (Roche) using SYBR Green I assays. The abundance of each candidate scaffold was normalized against the abundance of the *Hox* scaffold (scf7180001247533). The scaffold containing the *Hox* cluster was selected as the reference scaffold because it is a long scaffold that shows no significant difference in sequence coverage between the sexes, and so is almost certainly located on an autosome. Primers were designed with the Primer3 freeware with annealing temperatures of approximately 60°C and product sizes of about 200 bp [33]. Scaffold IDs and primer sequences are provided in S3 Table. Each reaction was carried out in triplicate, with a final concentration of each primer at 0.5 μM and total input of gDNA at 0.8 ng. The fluorescence crossing points (C_T_ values) were estimated with the second derivative maximum method using the LightCycler 1.5 software. PCR amplification efficiency was determined with a calibration curve for each primer pair, and the relative quantifications were carried out with amplification efficiency correction. Only PCR assays producing a single product (as verified by melting curve analysis) and with a PCR efficiency between 1.8 and 2.1 were included in subsequent analysis. The raw qPCR data is provided in S2 Table.

### Validation of Y-linked scaffolds

Candidate Y-linked scaffolds were validated by PCR on male and female genomic DNA samples, using ThermoPrime Plus DNA polymerase (Thermo Scientific). Double-distilled water was used as a template in the no template control. Primers were designed against unique regions of the candidate scaffold. Primer sequences are provided in S3 Table. Regions of each scaffold that are unique in the assembled genome were identified by a sliding window analysis: windows of 1 kb of sequence, at 0.5 kb offsets, were searched against the *Strigamia* reference genome using the NCBI program BLAST [34]. Windows that only returned hits to their constituent scaffold were selected for primer design. PCR products were visualized on 1% agarose gel with 0.1 ng/μl ethidium bromide. PCRs were run under the following conditions: an initial denaturation step of 95°C for 5 min; followed by 35 cycles of 95°C for 30 s, 58°C for 30 s, 72°C for 45 s; and finally an extension step of 72°C for 5 min.

### Production and digoxigenin labelling of the X-chromosome derived DNA probes

Unique regions on the validated X-linked scaffolds were identified by a sliding window analysis: 10 kb windows were searched against the *Strigamia* reference genome using the NCBI program BLAST [34]. Windows that only returned hits to their constituent scaffold were selected for primer design. PCR fragments were amplified using the Expand Long Template PCR System (Roche), according to the manufacturer’s instructions. Primer sequences are provided in S3 Table. PCR fragments were labelled by digoxigenin-11-dUTP (Roche) using the Nick Translation Kit (Abbott Molecular). The 25 μL labelling reaction contained 500 ng of purified PCR product, 1x nick translation buffer, 250 μM dATP, dCTP, and dGTP, 90 μM dTTP, 160 μM digoxigenin-11-dUTP and 5 μL of nick translation enzyme mix. The reaction mix was incubated for 7 h at 15°C. Each PCR fragment was labelled separately.

### Preparation of mitotic metaphase chromosome spreads from single *Strigamia* embryos

Individual eggs at embryonic stage 4–5 [for staging, see [31]] were partly dechorionated using fine tweezers in a physiological solution, and transferred into a hypotonic solution (0.065 M KCl) for 15–20 min. The eggs were then fixed in methanol/acetic acid (3:1) for 10 min. During fixation each embryo was removed from the remaining chorion and torn into about four pieces. Each piece of the embryo was transferred into a 1.5 μL drop of 60% acetic acid on a slide and spread on the slide using a heating plate at 55°C. For chromosome counts and karyotype analyses, some preparations were directly stained with 0.5 μg/mL DAPI (49,6-diamidino-2-phenylindole; Sigma-Aldrich) in antifade based on DABCO (1,4-diazabicyclo[2.2.2]octane; Sigma-Aldrich). The other preparations were passed through a graded ethanol series (70%, 80%, and 100%, 30 s each) and stored at −20°C until further use.

### Preparation of meiotic chromosome spreads

Meiotic chromosomes were obtained from the testes of sub-adult males as described in [35]. Briefly, after dissection in a physiological solution, the testes were incubated in a hypotonic solution (0.075 M KCl) for 10 min, fixed in Carnoy fixative (ethanol/chloroform/acetic acid, 6:3:1) for 10–15 min, dissociated with tungsten needles in a drop of 60% acetic acid and spread on the slide using a heating plate at 45°C. Then the preparations were passed through a graded ethanol series (see above) and stored at −80°C until further use.

### Comparative Genomic Hybridization (CGH)

Genomic probes were prepared from gDNA extracted separately from adult males and females by the standard phenol-chloroform procedure. The probes were labelled using a Nick Translation Kit (Abbott Molecular Inc.); male DNA with Cy3-dUTP (GE Healthcare) and female DNA with fluorescein-12-dUTP (Invitrogen). Unlabelled female gDNA, used as a species-specific competitor, was sonicated using a Sonopuls HD 2070 (Bandelin Electric), with two cycles of five pulses at 70% power. CGH was performed essentially following the procedure described in [9]. Briefly, chromosome preparations were removed from the freezer, dehydrated in an ethanol series, treated and denatured. Then the preparations were hybridized with a denatured probe cocktail containing labelled male and female gDNAs (250 ng each), unlabelled sonicated female gDNA (3 μg), and sonicated salmon sperm DNA (25 μg) for 3 days at 37°C, washed for 5 min at 62°C in 0.1x SSC containing 1% Triton X-100, counterstained with 0.5 μg/mL DAPI and mounted in antifade based on DABCO.

### Fluorescence in situ hybridization (FISH) with the X-chromosome derived DNA probes

For FISH, we followed the procedure described in [35] with some modifications. Briefly, slides were digested with 100 μg/mL RNase A to remove potential transcripts of target sequences, incubated in 10 mM HCl for 20 min in 37°C to reduce the amount of cytoplasm and blocked for 30 min in 5x Denhardt solution (1% Ficoll, 1% polyvinylpyrrolidone and 1% bovine serum albumin). Then, the chromosomes were denatured in 70% deionized formamide, 2x SSC for 3 min 30 s at 68°C. After denaturation, the slides were hybridized overnight with a probe cocktail denatured for 5 min at 90°C. The probe cocktail for one slide (10 μL) contained 40 ng of each labelled PCR fragment and 25 μg of sonicated salmon sperm DNA (Sigma-Aldrich) in 50% formamide, 10% dextran sulphate and 2x SSC. Hybridization signals were detected and amplified by a series of three antibodies, mouse anti-digoxigenin (1:100, Roche), sheep anti-mouse Ig conjugated with digoxigenin (1:200, Millipore), and sheep anti-digoxigenin conjugated with fluorescein (1:200, Roche). The preparations were counterstained with 0.5 μg/mL DAPI and mounted in antifade based on DABCO.

### Microscopy and Image Processing

Preparations were observed in a Zeiss Axioplan 2 microscope equipped with appropriate fluorescence filter sets. Black-and-white images were taken either with a cooled F-View CCD camera (DAPI- and CGH-stained preparations) or an Olympus CCD monochrome camera XM10, and captured separately for each fluorescent dye with either AnalySIS software, version 3.2 (Soft Imaging System GmbH) or with cellSens 1.9 digital imaging software (Olympus Europa Holding), respectively. The images were pseudo-coloured and merged using Adobe Photoshop CS4 and CS5 (Adobe Systems Inc.).

## Supporting Information

S1 Fig Comparative genomic hybridization on a spermatocyte pachytene complement prepared from the testes of a sub-adult male of *Strigamia maritima*. Chromosomes were counterstained with DAPI (blue). Female-derived genomic probe was labelled with fluorescein-12-dUTP (green) and male-derived genomic probe with Cy3-dUTP (red). Panels (**A**-**D**) show detailed analysis of the pachytene complement: (**A**) merged image; (**B**) DAPI image; (**C**) hybridization pattern of the female genomic probe; (**D**) hybridization pattern of the male genomic probe. Both probes highlighted one heterochromatic arm of the large metacentric bivalent (arrow), the nucleolus (N) associated with a middle-sized bivalent, and the centromeric heterochromatin of all chromosomes (asterisks), but did not differentiate a sex chromosome pair. Scale bar = 10 μm.
(TIF)

S2 Fig Validation of six male-specific scaffolds in the *Strigamia maritima* genome. Original, uncropped gels from Fig 2C. (**A-F**) correspond to the panels labelled 1 to 6 respectively, and (**G**) to the control panel, in Fig 2C in the main text.
(TIF)

S1 Table List of candidate and validated X- and Y-linked scaffolds in the *Strigamia maritima* genome. (XLSX)

S2 Table Raw data for Fig 2. Coverage and length of each genomic scaffold, and qPCR data to validate X-linked scaffolds.
(XLSX)

S3 Table PCR primer sequences used in this study. (XLSX)
