# Supplementary figures and images for: XX/XY System of Sex Determination in the Geophilomorph Centipede Strigamia maritima
Source: PLoS One. 2016 Feb 26;11(2):e0150292. doi: 10.1371/journal.pone.0150292 (PMC4769173; doi:10.1371/journal.pone.0150292)

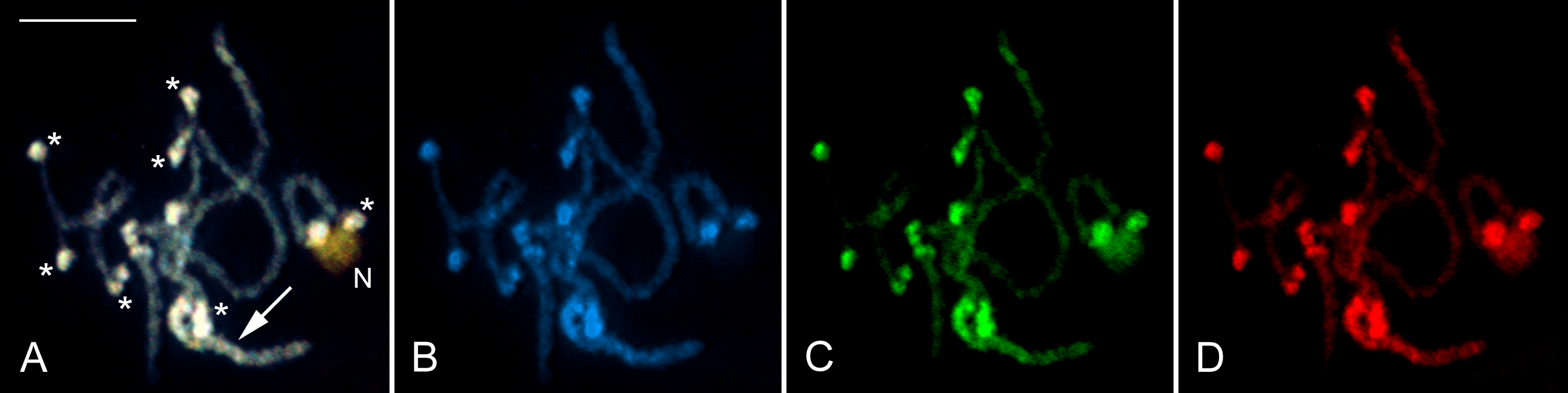

Supplement: S1 Fig — Chromosomes were counterstained with DAPI (blue). Female-derived genomic probe was labelled with fluorescein-12-dUTP (green) and male-derived genomic probe with Cy3-dUTP (red). Panels (A-D) show detailed analysis of the pachytene complement: (A) merged image; (B) DAPI image; (C) hybridization pattern of the female genomic probe; (D) hybridization pattern of the male genomic probe. Both probes highlighted one heterochromatic arm of the large metacentric bivalent (arrow), the nucleolus (N) associated with a middle-sized bivalent, and the centromeric heterochromatin of all chromosomes (asterisks), but did not differentiate a sex chromosome pair. Scale bar = 10 μm. (TIF) [file pone.0150292.s001.tif]

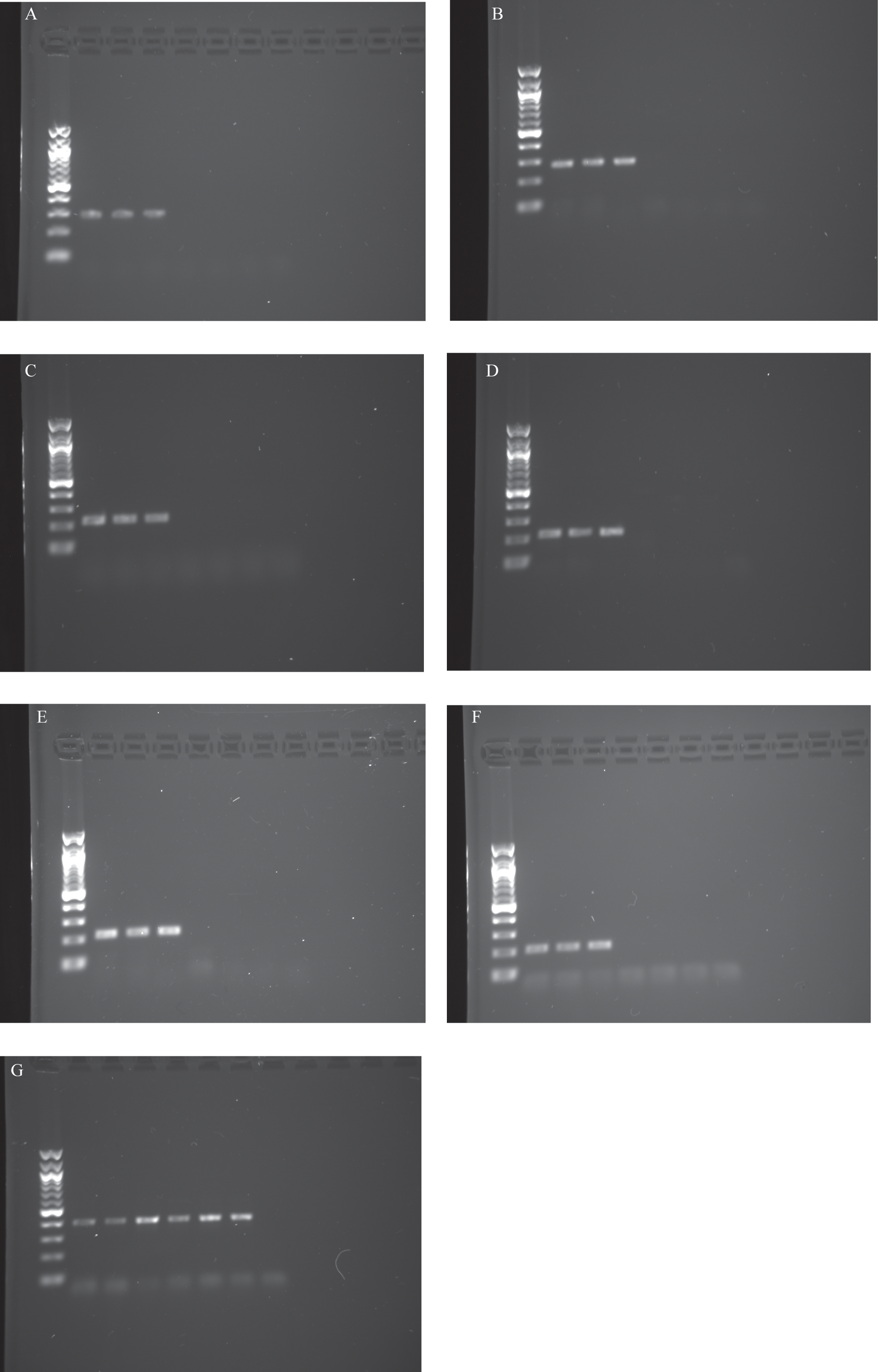

Supplement: S2 Fig — Original, uncropped gels from Fig 2C. (A-F) correspond to the panels labelled 1 to 6 respectively, and (G) to the control panel, in Fig 2C in the main text. (TIF) [file pone.0150292.s002.tif]
